# From visibility graphs to cognition

**DOI:** 10.3389/fnetp.2026.1830261

**Published:** 2026-05-29

**Authors:** Sabin Gautam, Paolo Grigolini

**Affiliations:** Department of Physics, University of North Texas, Denton, TX, United States

**Keywords:** diffusion entropy analysis, ergodicity breaking, meditation and cognition, network physiology, visibility graph

## Abstract

The aim of this study is to discuss the adoption of the popular natural visibility graph method (NVGM) to create complex networks and explore the condition, ignored by early work on this subject, where the dynamical system under analysis is characterized by a deviation from the ordinary ergodic condition, as a consequence of turbulent events (crucial events) with an inverse power law index μ ranging from 1 to ∞ . In the long-time limit for 2<μ<3 , the non-ergodic signal becomes virtually indistinguishable from the fractional Gaussian noise (FGN), hypothesized from the NVGM theory. To identify a genuine FGN, the method of statistical analysis, known as diffusion entropy analysis (DEA), with stripes is used. The adoption of DEA with stripes shows that the scaling δ is significantly reduced by meditation. The adoption of NVGM has the effect of generating a homogeneous network. This analysis yields a significant difference between sick and healthy patients. In the case of healthy patients, the adoption of stripes leaves the value of the scaling virtually unchanged, while for sick patients, the adoption of stripes yields a significantly lower value of δ , suggesting that their heartbeats host a FGN contribution. To explain the influence of meditation on δ , it is necessary to make the popular linear response theory by Kubo and his quantum mechanical prescription compatible with ergodicity breaking, using a master equation approach (MEA). The non-Markov MEA hosts ergodicity-breaking crucial events, leading to establish a connection with the literature on macroscopic effects of quantum mechanics. About the paradoxical effect of meditation-induced scaling reduction, it is suggested that more attention should be devoted to the statistical analysis of physiological processes after meditation.

## 1 Introduction

The natural visibility graph method (NVGM) illustrated by Lacasa et al. (2008) has recently been applied to shed light on various issues such as the concept of network complexity (Masoomy et al., 2023; Zhan and Xiao, 2024; Li et al., 2022; He et al., 2024; Gao and Ge, 2024; Schmidt and Köhne, 2023; Moreira et al., 2022; Hu and Xiao, 2022; Varley and Sporns, 2022; Gonçalves et al., 2016; Luque et al., 2009; Xie and Zhou, 2011; Averty et al., 2025). These studies explore a wide range of conditions, including information transport and scaling detection as evaluation of H , known as the Hurst coefficient, as discussed in Section 2. Another recent application of the NVGM (Lacasa et al., 2008) is the interpretation of the phenomenon of meditation (Nasrolahzadeh et al., 2023; Nasrolahzadeh et al., 2019). These two studies attracted the attention of many researchers to focus their attention to the papers (Nasrolahzadeh et al., 2023; Nasrolahzadeh et al., 2019) claiming a cure for Alzheimer’s disease (Nasrolahzadeh et al., 2025a; Vicchietti et al., 2023; Nasrolahzadeh et al., 2025b). The articles on meditation (Nasrolahzadeh et al., 2023; Nasrolahzadeh et al., 2019) are based on the analysis of the heart beats of the patients doing meditation. An important purpose of the analysis of heart beats is to find a way to distinguish healthy patients from patients with a high risk of heart attack. This important issue was discussed in 1999 by the Boston group (Havlin et al., 1999). The authors found that long-range correlations and multifractality are weaker in the case of heart failure. The same issue was addressed by Allegrini et al. (2002), who found that healthy individuals are characterized by a higher level of *memory beyond memory*, an important property that seems to be incompatible with the adoption of the NVGM (Lacasa et al., 2008), originally proposed to study fractional Gaussian noise (FGN) and fractional Brownian motion (FBM).

In this study, we aim to demonstrate that the open questions in the recent literature on meditation necessitate revisiting the arguments developed in the 1960s to either question or justify Kubo’s linear response theory (LRT) (Kubo, 1966). To explain the main goal of this study, it is convenient to stress that the stationary correlation function of FGN is characterized by the property $C\left(t\right)\propto \frac{1}{t^{2-2H}}$ . Note that for H>0.5 , this correlation function is not integrable, and the correlation time of the process is infinite. This condition is referred to, in this study, as “infinite memory” or “complexity of FGN.”

There exists another form of memory generated by turbulent phenomena that are described by Manneville (1980), with the power index z . The time distance between two consecutive turbulent events, denoted in this study as crucial events, is given by the waiting time distribution $\psi \left(\tau \right)\propto \frac{1}{\tau^{\mu }}$ with $\mu =\frac{z}{z-1}$ . The survival probability has the form $\Psi \left(\tau \right)\propto \frac{1}{\tau^{\mu -1}}$ , making it not integrable for μ<2 . The LRT used in this study to discuss the effects of meditation on heartbeats, as discussed in Section 2, is based on a rate of crucial event production that requires an infinite time to reach the stationary condition. This is defined as the system’s memory.

Section 2 establishes a connection between the system’s memory and its response to external perturbations, moving beyond traditional LRT, which assumes stationary equilibrium conditions. Section 3 illustrates the combined use of the NVGM and diffusion entropy analysis (DEA) to analyze heart rate patterns in healthy versus pathological cases. Section 4 illustrates a quantum-like approach to reconcile ergodicity breaking with non-Markov master equations. Finally, Section 5, stressing the importance of network physiology, discusses the conclusion and makes a proposal for future studies that should be conducted to establish a connection between criticality and cognition.

## 2 Memory-like property

To describe the research plan of this study, it is first necessary to recall the meaning of the stationary correlation function. The correlation function $\left\langle \xi \left(t_{1}\right)\;\xi \left(t_{2}\right)\right\rangle$ , in principle, depends on both $t_{1}$ and $t_{2}$ , while FGN (Mandelbrot and Van Ness, 1968) is based on the property shown in Equation 1:

$$\left\langle \xi \left(t_{1}\right)\;\xi \left(t_{2}\right)\right\rangle =C\left(\left|t_{1}-t_{2}\right|\right),$$ (1)

depending only on the modulus of the difference between two times. This is the definition of the stationary condition.

The method proposed by Allegrini et al. (2002), devoted to the analysis of heart beats, analyzes the sequence of time distances between the ith and the (i+1)th pulse, denoted by the symbol $T_{i}$ . The value $T_{i}$ , expressed as a function of i for very large i , can be regarded as a function ξ(t) , namely, as a function of a continuous time variable t , which represents the signal to be analyzed using the method of Allegrini et al. (2002). The vertical axis of the trajectory ξ(t) is divided into many stripes of finite size, and Allegrini et al. (2002) aimed to evaluate the time spent by the trajectory within the same stripe. The transition from one stripe to another is called *event*. Some of the events are proven to be renewal, namely, to be crucial events, and they can be detected using DEA, assuming that the value of scaling is δ>0.5 . To evaluate the scaling δ , we must study the trajectory $x\left(t\right)=\int_{0}^{t}\xi \left(t^{\prime }\right)\;dt^{\prime }$ expected to fulfill the renormalization group property (Goldenfeld, 2018) $P\left(x,t\right)=\frac{1}{t^{\delta }}F\;\left(\frac{x}{t^{\delta }}\right)$ .

Note that the time distance between two consecutive crucial events has the following waiting distribution density (Equation 2):

$$\psi \left(t\right)=\left(\mu -1\right)\;\frac{T_{C}^{\mu -1}}{{\left(T_{C}+t\right)}^{\mu }},$$ (2)

with μ>1 . It is important to emphasize that the theoretical origin of this inverse power law waiting time distribution density is Kolmogorov turbulence, as widely discussed by Ignaccolo et al. (2001). The crucial events are renewal and generate ergodicity breaking and non-stationary correlation functions. The readers can find a discussion of this issue in the studies of Zumofen and Klafter (1993), Hou et al. (2018), Metzler et al. (2014), and Lubelski et al. (2008).

However, when the condition μ>2 applies, as discussed by Allegrini et al. (2003), the non-stationary correlation functions become stationary in the long-time limit. To avoid confusion with other forms of ergodicity breaking (Bernaschi et al., 2020; Roy and Lazarides, 2020; Teeriaho et al., 2025), it is convenient to stress that this study focuses on weak ergodicity breaking (Bel and Barkai, 2005).

It is important to note that $T_{C}$ is a short time introduced to go beyond the statement that $\psi \left(\tau \right)\propto \frac{1}{\tau^{\mu }}$ is the proper definition of waiting distribution density of crucial events. This waiting time distribution density is normalized, and it does not diverge at τ=0 . The corresponding survival probability Ψ(τ) at τ=0 fits the normalization condition Ψ(0)=1 . In the case of the FGN correlation function, the role of $T_{C}$ is played by a time called $T_{B}$ , as shown in Equation 6.

To evaluate the scaling numerically in the case of weak ergodicity breaking, we generate a trajectory x(t) that is constant between two events and makes a jump ahead of intensity W when an event occurs. According to Grigolini et al. (2001), the DEA applied to the analysis of the stochastic trajectory x(t) leads to Equation 3:

$$S\left(t\right)=A+\delta \ln t.$$ (3)

The resulting scaling $\delta =\frac{1}{\mu -1}>0.5$ is obtained even if some events are not crucial. In fact, non-crucial events would yield a scaling δ=0.5 and, in the long-time limit DEA, perceives only the events generating larger scaling.

Crucial events are expected to generate non-stationary correlation function (Allegrini et al., 2003). However, according to Geisel et al. (1985), under the condition $t_{1}\neq t_{2}$ and 2<μ<3 , Equation 4 can be obtained:

$$\frac{\left\langle \xi \left(t_{1}\right)\;\xi \left(t_{2}\right)\right\rangle }{\left\langle \xi^{2}\right\rangle }=\frac{1}{\left\langle \tau \right\rangle }\int_{t_{1}-t_{2}}^{\infty }\;\left(\tau -|t_{1}-t_{2}|\right)\;\psi \left(\tau \right)d\tau \;,$$ (4)

with $<\tau >=T_{C}/\left(\mu -2\right)$ , which, in the long-time limit, yields Equation 5:

$$\frac{\langle \xi \left(t_{1}\right)\;\xi \left(t_{2}\right)\rangle }{\langle \xi^{2}\rangle }={\left(\frac{T_{C}}{T_{C}+|t_{1}-t_{2}|}\right)}^{\mu -2},$$ (5)

namely, a stationary correlation function.

It is important to emphasize that according to Mandelbrot and Van Ness (1968), the correlation function of an FGN is presented in Equation 6:

$$\frac{\langle \xi \left(t_{1}\right)\;\xi \left(t_{2}\right)\rangle }{\langle \xi^{2}\rangle }=\Phi_{\xi }^{\left(FGN\right)}\left(t_{1},t_{2}\right)={\left(\frac{T_{B}}{T_{B}+|t_{1}-t_{2}|}\right)}^{\eta },$$ (6)

with η=2−2H , where H represents the Hurst coefficient to denote the scaling in a condition that lacks crucial events. Since the NVGM is used to study FGN, the comparison between Equations 5, 6 allows us to establish which values of H emerges from crucial event, yielding Equation 7:

μ−2=η=2−2H, (7)

leading to Equation 8:

$$H=\frac{4-\mu }{2}.$$ (8)

Equation 8 shows that for 2<μ<3 , a complex process becomes indistinguishable from FGN, with scaling index H>0.5 , a process with stationary correlation function not hosting crucial events. The application of LRT to the crucial events generates the same results as the LRT applied to FGN. To prove this important property, let us first apply LRT (Kubo, 1966) to FGN. According to LRT, the response of a variable $\xi_{S}\left(t\right)$ to a perturbation $\xi_{P}\left(t^{\prime }\right)$ is presented in Equation 9:

$$\left\langle \xi_{S}\left(t\right)\right\rangle =\varepsilon \int_{0}^{t}\;\chi \left(t,t^{\prime }\right)\;\xi_{P}\left(t^{\prime }\right)dt^{\prime },$$ (9)

where $\xi_{P}\left(t^{\prime }\right)$ is the perturbing signal. Note that the adoption of $\left\langle \xi_{S}\left(t\right)\right\rangle$ in LRT represents the quantum mechanical mean value, while in our approach, $\left\langle \xi_{S}\left(t\right)\right\rangle$ represents the result of an average over many distinct trajectories. The function $\chi \left(t,t^{\prime }\right)$ is called linear susceptibility. In the case where $\xi_{S}$ , in the absence of perturbation, is FGN, the linear susceptibility is presented in Equation 10 (Hänggi and Thomas, 1982):

$$\chi \left(t,t^{\prime }\right)=\frac{d}{dt^{\prime }}\;\phi_{\xi }\left(t,t^{\prime }\right)=\frac{d}{dt^{\prime }}{\left[\frac{T_{B}}{T_{B}+|t-t^{\prime }|}\right]}^{\eta },$$ (10)

yielding Equation 11:

$$\chi \left(t,t^{\prime }\right)=\eta \;\frac{T_{B}^{\eta }}{{\left[T_{B}+|t-t^{\prime }|\right]}^{\eta +1}}.$$ (11)

In this case, the linear susceptibility is a stationary property, reflecting the stationary nature of the corresponding correlation function. Let us now study the application of LRT to systems hosting crucial events for 2<μ<3 . Moreover, in this case, in the long-time limit of Equation 5, the correlation function of ξ is stationary; even if in the absence of perturbation, ξ hosts crucial events. The prescription of Aquino et al. (2010) yields Equation 12:

$$\chi \left(t,t^{\prime }\right)=R\left(t\right)\;\Psi \left(t-t^{\prime }\right).$$ (12)

R(t) is the rate of crucial events, as shown in Equation 13:

$$R\left(t\right)=\frac{1}{\left\langle \tau \right\rangle }\left[1+{\left(\frac{T_{C}}{T_{C}+t}\right)}^{\mu -2}\right],$$ (13)

with Equation 14

$$\left\langle \tau \right\rangle =\frac{T_{C}}{\mu -2},$$ (14)

for 2<μ<3 . As a consequence, the linear susceptibility $\chi \left(t,t^{\prime }\right)$ is not stationary. However, in the asymptotic limit, as t→∞ , we can obtain Equation 15:

$$R\left(t\right)\approx \frac{1}{\left\langle \tau \right\rangle }$$ (15)

and Equation 16:

$$\chi \left(t,t^{\prime }\right)=\frac{1}{\left\langle \tau \right\rangle }{\left(\frac{T_{C}}{T_{C}+\left(t-t^{\prime }\right)}\right)}^{\mu -1},$$ (16)

namely, the linear susceptibility becomes stationary again. The assumption that Equations 11, 16 generate the same physical decay (Equation 17):

η+1=μ−1, (17)

using again η=2−2H , recovers Equation 7 and the Hurst coefficient H of Equation 8. Note that the NVGM (Lacasa et al., 2008) was originally introduced to study FGN and FBM. FBM is non-stationary but is obtained from the integration of FGN, a stationary process.

## 3 Visibility graph

**FIGURE 1:**
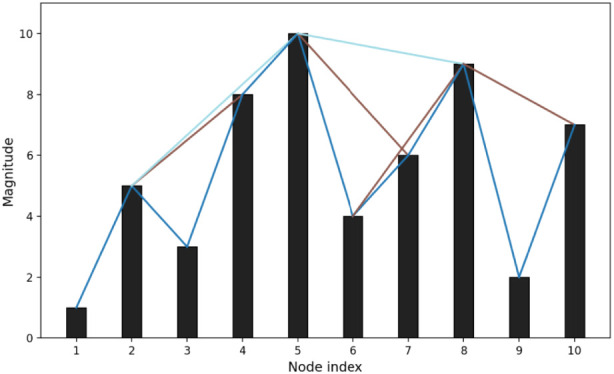
Illustration of the visibility graph construction. Each vertical bar represents the magnitude of an individual time series value, referred to as a node. Continuous lines indicate visibility-based connections between nodes. If a taller bar (a node with greater magnitude) lies between two non-adjacent nodes, it obstructs their line of sight, and no connection is formed between them.

After showing that, in the long-time limit, crucial events for 2<μ<3 yield a stationary condition, we now discuss the NVGM algorithm. This algorithm, as shown in Figure 1, transforms a time series into a network by interpreting each data point as a node and connecting two nodes if they are “visible” to each other with the following prescription shown in Equation 18 (Lacasa et al., 2008):

$$y_{c}<y_{b}+\left(y_{a}-y_{b}\right)\;\frac{t_{b}-t_{c}}{t_{b}-t_{a}}.$$ (18)

According to Lacasa et al. (2008), in fractal signals such as FBM, prominent peaks block visibility over long-time intervals, leading to heavy-tailed degree distributions. Different types of time series give rise to distinct graph structures: fractal series become scale-free graphs, random series form random graphs, and periodic series lead to regular graphs. The visibility graph of FBM is scale-free, a property widely exploited in fractal/physiological time-series analyses (Lacasa et al., 2009; Lacasa et al., 2008). When a fractal time series is converted into a graph, the resulting node degree distribution (P(k)) exhibits the following scale-free distribution, as shown in Equation 19 (Lacasa et al., 2009), (Li et al., 2016), (Nasrolahzadeh et al., 2023):

$$P\left(k\right)\propto k^{-\gamma \left(H\right)},$$ (19)

with Equation 20:

$$\gamma \left(H\right)=3-2H,$$ (20)

involving the Hurst index again because Lacasa et al. (2009), Li et al. (2016), and Nasrolahzadeh et al. (2023) apply the NVGM to both FGN and FBM. Note that k represents the node degree. This reflects the existence of a quantitative relationship between the exponent γ of the power-law degree distribution in the visibility graph and H . Consequently, the visibility algorithm serves as an effective alternative for estimating H , along with μ , when the condition of Equation 8 applies. Validation studies (Ni et al., 2009; Lacasa et al., 2009) using artificial FBM with known Hurst exponents consistently demonstrate high correlation coefficients $\left(R^{2}>0.97\right)$ between theoretical predictions and empirical measurements. FGN, defined as the increments of a FBM,

$$\Delta B_{H}\left(t\right)=B_{H}\left(t+1\right)-B_{H}\left(t\right),$$

has a power spectrum S(f) that behaves as follows (Addison, 1997):

$$S\left(f\right)∼\frac{1}{f^{\beta }},$$

with Equation 21:

β=2H−1. (21)

As a consequence γ(β)=4−β , yielding for FGN, we can obtain Equation 22:

$$\gamma \left(H\right)=5-2H$$ (22)

rather than Equation 20.

**FIGURE 2:**
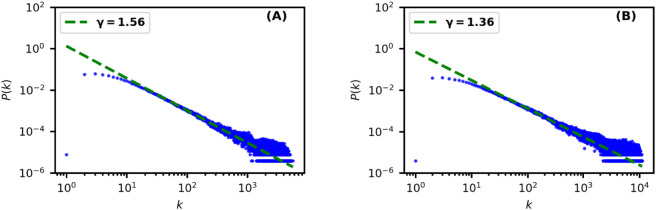
Node degree distribution of NVGM applied to surrogate sequences generated by Mandelbrot and Van Ness (1968) with H=0.7 **(A)** and H=0.8 **(B)**. The node degree distribution follows a power law: $P\left(k\right)∼k^{-\gamma \left(H\right)}$ .The fitted exponents γ=1.56 **(A)** and γ=1.36 **(B)**, consistent with the theoretical prediction γ=3−2H .

Before moving on to our main results concerning FGN, let us review the approach adopted by Lacasa et al. (2008) and Lacasa et al. (2009) to discuss the FBM processes. This will make it easier for the readers to understand our use of NVGM to deal with crucial events. To confirm the proposed relationship between γ and H (Lacasa et al., 2009), the Mandelbrot algorithm (Mandelbrot and Van Ness, 1968) is used to generate artificial FBM series of $10^{5}$ data with H=0.7 and H=0.8 . For surrogate FBM series, it might be difficult to accurately estimate the power law exponent γ from log–log plots due to inherent biases and finite-size effects. The approach proposed by Clauset et al. (2009), known as maximum likelihood estimation (MLE), is applied here. This is a more robust method for determining the exponent, especially when the visibility graph exhibits scale-free behavior, and is presented in Equation 23:

$$\gamma \left(H\right)=1+n{\left[\overset{n}{\underset{i=1}{\sum }}\ln\left(\frac{k_{i}}{k_{\min}}\right)\right]}^{-1},$$ (23)

where $k_{i}$ and i=1,2,…,n are the observed values of the node degree k and $k_{\min}$ is the lower bound above which the power-law behavior is assumed to be hold. Li et al. (2016), more recently than the original study of Lacasa et al. (2009), assume that the first dip (or local minimum) in the Kolmogorov–Smirnov statistics often indicates the optimal $K_{\min}$ , marking it the point where the model begins to fit the data well. However, in the case of FBM or other fractal-like long time series, it is common (Lacasa et al., 2009) to use a fixed value of $K_{\min}\approx 10$ , allowing for a typical variation of approximately 5% . Using γ(H)=3−2H to analyze FBM yields the results illustrated in Figure 2, which are remarkably similar to those of Figure 2 of the study by Lacasa et al. (2009), thus confirming the proper use of the NVGM.

Now, we address the challenging issue of applying the NVGM to real-world electrocardiogram (ECG) data obtained from PhysioNet for 15 Healthy subjects (Nemcova et al., 2020) and 19 subjects with congestive heart failure (CHF) (Martin-Yebra et al., 2024). These continuous 24-h ECG recordings were acquired at a high-resolution sampling frequency of 1,000 Hz. ECG signals were band-pass filtered between 0.5– 40Hz using a fourth-order Butterworth filter applied in a zero-phase manner to reduce baseline drift and high-frequency noise. R-peaks were identified using an adaptive amplitude threshold, with a minimum inter-peak interval of 0.3 s , to ensure physiologically plausible detections, which is consistent with the classical QRS detection principles described by Pan and Tompkins (1985). RR intervals were calculated from successive R-peaks, and DEA with and without stripe was used to find δ . We adopt the methodology proposed in the recent publication of Schizas et al. (2025), who discuss in detail the choice of a stripe size for the ECG signal and the procedure for the automated selection of the linear fitting region in the DEA plot.

**FIGURE 3:**
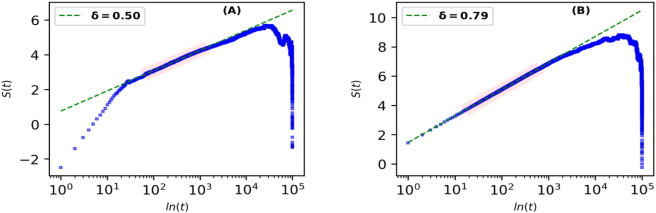
Comparison between the use of DEA with stripes **(A)** and without stripes **(B)** for FGN generated using surrogate sequences (Mandelbrot and Van Ness, 1968).

According to Culbreth et al. (2019), the adoption of DEA with stripes has the effect of killing the scaling complexity generated using FGN. Figure 3 confirms the arguments of Culbreth et al. (2019). The DEA was applied to an FGN process, with H=0.8 . In the left panel of Figure 3, DEA with stripes yields δ=0.5 , whereas in the right panel, DEA without stripes yields δ=H=0.79 . This confirms the prediction of Culbreth et al. (2019) that DEA with stripes annihilates the complexity of FGN.

This interesting result leads us to point out that FGN can be generated by a pathology killing crucial events with no significant reduction in scaling. Benfatto et al. (2021) studied the bio-photons emitted by a set of seeds watered to stimulate the germination process. Germination is a form of phase transition, generating crucial events and ergodicity breaking. However, when seeds are grown in an environment that lacks the necessary life condition, their complexity remains high, but crucial events are lost, and the use of DEA with stripes produces a significant reduction in scaling δ .

Jelinek et al. (2021) studied the heartbeats of patients under the influence autonomic neuropathy of increasing severity for the main objective of establishing whether the increasing severity of the disease has the effect of turning 1/f noise, which is conjectured to be a sign of healthy condition, into white noise. They found that the highest severity does not have this effect, but it turns the 1/f noise generated by crucial events into the 1/f noise generated by FGN.

**FIGURE 4:**
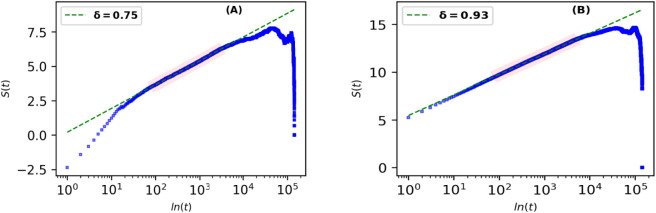
Diffusion entropy analysis: S(t) as a function of ln(t) for the RR time series of a CHF subject. **(A)** DEA with stripes and **(B)** DEA without stripes.

**FIGURE 5:**
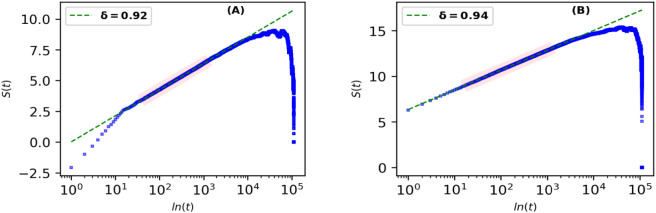
Diffusion entropy analysis: S(t) as a function of ln(t) for the RR time series of a healthy subject. **(A)** DEA with stripes and **(B)** DEA without stripes.

The findings of Benfatto et al. (2021) and Jelinek et al. (2021) lead us to the conclusion that the RR signals of the sick patients correspond to a superposition of crucial events and FGN and that their high complexity, with δ=0.93 , is due to FGN, thereby causing DEA with stripes to yield a strong reduction, which, as shown in Figure 4, turns δ=0.93 into δ=0.75 . The statistical analysis of the healthy patients in Figure 5, with a significant component of crucial events, yields a significantly different result. The adoption of DEA with stripes changes δ=0.94 into δ=0.92 , which is a much smaller complexity reduction.

**FIGURE 6:**
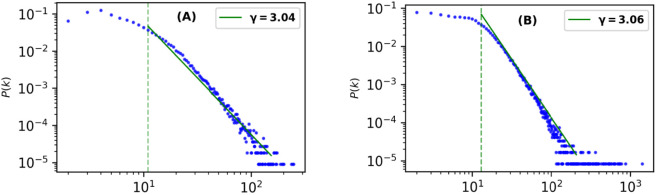
Node degree distribution as a function of node degree obtained from NVGM of RR interval time series for a healthy subject **(A)** and a CHF subject **(B)**.

The main conclusion of our use of NVGM is that it is extremely difficult to differentiate FGN from crucial events when the condition 2<μ<3 applies. As a consequence, a large part of the research work based on NVGM may deal with processes hosting crucial events, making it difficult to establish a distinction between healthy and sick physiological conditions. Figure 6 shows that in both healthy and pathological cases, H scaling is equivalent to δ scaling obtained from DEA without stripes. Using the approach of Allegrini et al. (2002), based on the adoption of stripes, kills the complexity of FGN, making it possible to distinguish FGN from crucial events. This allows us to establish the contribution of FGN to the complexity of the process under study. This approach is expected to improve the efficiency of NVGM for detection and prediction of heart failure.

**FIGURE 7:**
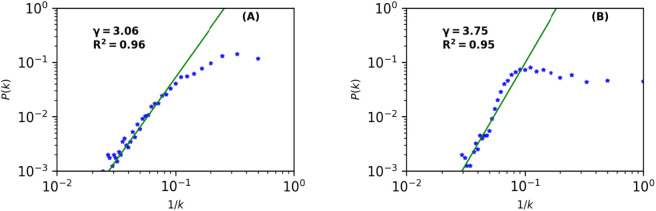
Node degree distribution as a function of node degree obtained from NVGM of RR interval time series for subject-1 before **(A)** and during **(B)** Chi-meditation.

Another important result of our use of NVGM is that this technique leads to recovering a surprising effect of meditation already found in the earlier work of Tuladhar et al. (2018). To demonstrate this important effect of meditation, we refer to Figure 7. The interesting findings of Nasrolahzadeh et al. (2023) was recovered, showing that meditation has the effect of increasing the power-law exponent γ , through the relation $H=\frac{5-\gamma }{2}$ . This corresponds to a decrease in the Hurst coefficient H . Supplementary Table S3 of a Supplementary Material refers to real RR data under the influence of Chi-meditation. This illustrates and confirms the same property observed in meditation-induced γ increase. This is an effect closely connected to the decrease in scaling δ observed in the earlier work of Rohisha et al. (Tuladhar et al., 2018), in Yoga and Chi meditation. Figure 7, along with Figures 8, 9 of Nasrolahzadeh et al. (2023), referring to real RR sequence data, demonstrates that meditation has the effect of increasing the index γ in the scale distribution $p\left(k\right)=C/k^{\gamma }$ . The main result of Section 2, namely that in the long-time limit processes hosting crucial events with 2<μ<3 become indistinguishable from FGN processes, suggests that the reduction of H as an effect of meditation is, actually, for healthy individuals, a reduction of the scaling δ .

The connection between scaling and intelligence is widely conjectured. Refer to the study by Linkenkaer-Hansen et al. (2001), which has an impressively large number of quotations. A special attention should be devoted to Müller et al. (2025), stressing the connection between criticality and cognition. Cognitive performance is generated by criticality, and the power-law scaling established using NVGM affects the low-frequency range of the power spectrum (Fujimoto et al., 2025). These concepts are used to study Alzheimer’s disease (Javed et al., 2025), which is generated, according to a plausible conjecture, using the physiological processes in which a patient transitions from criticality to super-criticality. Xin et al. (2025) interpreted criticality as a genetic property. The same theoretical perspective is used to explain Parkinson’s disease (Lee et al., 2025), and criticality is also discussed as a form of metastability (Hancock et al., 2025).

Strogatz (2001), Watts and Strogatz (1998), and Albert et al. (2000) concluded that the weak ergodicity breaking is compatible with the emergence of the scale-free distribution $p\left(k\right)=C/k^{\gamma }$ . However, the concept of network complexity to explain the effects of meditation is not helpful. The use of NVGM shows that the meditation-induced increase of γ is nothing but the reduction of δ shown by Tuladhar et al. (2018), suggesting that meditation favors randomness, despite the claim that the scale-free distribution of links makes the complex system more robust (Strogatz, 2001).

## 4 Quantum-like interpretation

The quantum-like approach discussed in this section contributes to clarify the role of crucial events discussed in the earlier section. Consider two quantum states |1〉 and |2〉 and let us adopt the non-Markov prescription proposed by Barbi et al. (2005), as presented in Equation 24:

$$\frac{d}{dt}P\left(t\right)=-\int_{0}^{t}d\tau \Phi \left(t-\tau \right)KP\left(\tau \right),$$ (24)

where (Equation 25)

$$P\left(t\right)=\left(\begin{array}{c} p_{1}\left(t\right)\\ p_{2}\left(t\right) \end{array}\right),$$ (25)

with $p_{1}\left(t\right)$ and $p_{2}\left(t\right)$ denoting the probability of states |1〉 and |2〉 , respectively, leading to the normalization condition shown in Equation 26:

$$p_{1}\left(t\right)+p_{2}\left(t\right)=1.$$ (26)

The matrix K is defined in Equation 27:

$$K\equiv \frac{1}{2}\left(\begin{array}{cc} 1 & -1\\ -1 & 1 \end{array}\right),$$ (27)

which, under the Markov condition, corresponds to the structure of a conventional master equation.

Barbi et al. (2005) used the non-Markov prescription of Equation 24 to establish a connection with the celebrated proposal by Montroll and Weiss (1965), known as the Continuous Time Random Walk (CTRW). As explained by Allegrini et al. (2003), the time evolution of P(t) is presented in Equation 28:

$$P\left(t\right)=\overset{\infty }{\underset{n=0}{\sum }}\int_{0}^{t}\psi_{n}\left(t^{\prime }\right)\Psi \left(t-t^{\prime }\right)dt^{\prime }M^{n}P\left(0\right),$$ (28)

where (Equation 29)

$$M\equiv \left(\begin{array}{cc} 1/2 & 1/2\\ 1/2 & 1/2 \end{array}\right).$$ (29)

It is important to highlight that CTRW in Equation 24 is connected with the crucial events of the earlier sections. In fact, $\psi_{n}\left(t\right)$ is the probability that n crucial events occurred and that the last event took place at time t . This yields Equation 30:

$$\psi_{n}\left(t^{\prime }\right)=\int_{0}^{t}\psi_{n-1}\left(t-t^{\prime }\right)\psi_{1}\left(t^{\prime }\right)dt^{\prime },$$ (30)

where $\psi_{1}\left(t\right)=\psi \left(t\right)$ of Equation 2. Following Allegrini et al. (2003), we must set the condition I−M=K .

This condition using the Laplace transform and the Convolution theorem leads to Equation 31:

$$\hat{\Phi }\left(s\right)=\frac{s\hat{\psi }\left(s\right)}{1-\hat{\psi }\left(s\right)},$$ (31)

where $\hat{\Phi }\left(s\right)$ is the Laplace transform of Φ(t) and $\hat{\psi }\left(s\right)$ is the Laplace transform of ψ(t) .

Allegrini et al. (2005) demonstrated that for μ>2 , the CTRW in Equation 28 becomes compatible with the stationary properties, making it possible to benefit from the use of visibility graph. Section 3 shows that the main effect of meditation is not to make the complex network generated by NVGM more complex. Meditation has the main effect of decreasing the value of the scaling parameter δ . The condition μ<2 , on the contrary, generates a perennial out of equilibrium condition that makes even more difficult to understand the effects of meditation.

Section 4 of Supplementary Material shows that Equation 9 can be written as Equation 32:

$$\left\langle \xi_{S}\left(t\right)\right\rangle =\varepsilon \int_{0}^{t}dt^{\prime }\;\chi \left(t,t^{\prime }\right)\;\xi_{P}\left(t^{\prime }\right)=\varepsilon \int_{0}^{t}dt^{\prime }\;\chi \left(t,t^{\prime }\right)\;f\left(t^{\prime }\right).$$ (32)

This is an important result of this study, replacing the stationary linear susceptibility $\chi \left(t,t^{\prime }\right)$ of the traditional LRT prescription based on quantum mechanics with the non-stationary susceptibility $\chi \left(t,t^{\prime }\right)=\frac{d}{dt^{\prime }}C\left(t,t^{\prime }\right)$ . This linear susceptibility is not stationary because $C\left(t,t^{\prime }\right)=\left\langle \xi \left(t\right)\;\xi \left(t^{\prime }\right)\right\rangle$ is the non-stationary correlation function (Equation 33):

$$C\left(t,t^{\prime }\right)=\int_{0}^{t^{\prime }}R\left(t^{\prime \prime }\right)dt^{\prime \prime }\langle \exp\left(-\int_{t^{\prime }}^{t}r\left(\tau \right)\;d\tau \right)\;\rangle .$$ (33)

On the other hand, Section 4 of the Supplementary Material yields Equation 34:

$$C\left(t,t^{\prime }\right)=\int_{0}^{t^{\prime }}R\left(t^{\prime \prime }\right)\;\Psi \left(t-t^{\prime \prime }\right)dt^{\prime \prime },$$ (34)

and proves that $C\left(t,t^{\prime }\right)$ in the long-time limit becomes identical to the stationary correlation function of Equation 5.

## 5 Conclusions

This final section is divided into six subsections to facilitate understanding of the results.

### 5.1 NVGM and crucial events

The first part of this paper, Sections 2 and 3, refers to the combined use of NVGM and DEA, with and without stripes, for the main purpose of evaluating the risk of heart failure. An earlier work on this important issue was conducted by Allegrini et al. (2002), concluding that scaling of pathological patients remained close to the high values of healthy patients, but the concentration of crucial events was found to be much smaller for pathological individuals.

This earlier work, however, did not analyze the real nature of the large concentration of non-crucial events of sick patients. Culbreth et al. (2019) made the plausible conjecture that the high concentration of non-crucial events may be because the experimental signal on heartbeats may be a superposition of healthy crucial events and FGN. Figure 4 and Supplementary Table S2 of the Supplementary Material of this paper fully confirm this conjecture. The new datasets (Nemcova et al., 2020; Martin-Yebra et al., 2024) leading to this important result were generated in 2024, while the data analyzed in the earlier work of Allegrini et al. (2002) date back to 2000.

As far as meditation is concerned, the main conclusion of the first part of this paper is that the combined use of NVGM and DEA, with and without stripes, proves that meditation has the effect of reducing the scaling value δ . This is a paradoxical result that should be settled by future research work.

### 5.2 Linear response theory

LRT, although very popular, has generated some controversial issues. A well-known criticism comes from Van Kampen (1971), who criticized microscopic linearity. The arguments of van Kampen have been discussed by Van Vliet (1988), who proved that lRT prediction remains widely reliable for the distribution of the unstable single trajectories. For the main conclusions of this paper, Hänggi and Thomas (1982) proved that the linear susceptibility of LRT is the time derivative of the equilibrium correlation function of the system before perturbation.

The discovery that complex networks with dynamical properties based on the use of CTRW (Sokolov et al., 2001) hosting crucial events generate non-stationary correlation functions led Sokolov and Klafter (2006) to claim that the ergodicity breaking is incompatible with linear response to weak perturbation. They claim, in fact, that this can be perceived as the death of the LRT of Kubo. We support the opposite claims of Aquino et al. (2011), in line with the view of Aquino et al. (2010), that LRT survives the occurrence of ergodicity breaking. These authors based their reply on a comparison between the phenomenological and dynamical approaches to LRT. Both approaches are in the spirit of Kubo’s LRT and lead to the conclusion that a perturbation hosting crucial events with exponent μ(P)<μ(S) , where P denotes the perturbation, can transmit its complexity to the perturbed system S .

The stochastic approach to quantum-like model of Section 4 has the attractive property of making the quantum-like formalism compatible with the ergodicity breaking induced by crucial events. It is important to note that Fonseca and Grigolini (1986), based on the adoption of the Fokker–Planck formalism, obtained results identical to those of Devoret et al. (1984), which are currently interpreted as macroscopic quantum-mechanical manifestations.

Finally, to establish a connection with Section 3, it is important to note that Tuladhar et al. (2017) found that FGN may be derived using a quantum-like formalism that generates a slowly decaying but stationary correlation function proportional to $1/\tau^{\eta }$ , where η=2−2H .

### 5.3 Quantum mechanics

Whether or not a quantum system may host crucial events is an issue not yet properly settled, thereby generating confusing conclusions. Note the important contributions of Deco et al. (2025a) and Deco et al. (2025b). In fact, these papers can be interpreted as connected to the occurrence of crucial events in quantum mechanics. The study of Deco et al. (2025a) is an interesting example of recent attempts made to make quantum mechanics compatible with criticality. In a further study (Deco et al., 2025b), the same group signals the emergence of contribution to criticality “revealing the necessary non-local (but non-quantum) properties of the human brain.” It would be interesting to assess whether this contribution is related to the “quantum-like” properties illustrated in Section 4 of this paper.

To support our interpretation of quantum systems hosting crucial events, it is convenient to quote the two additional studies of Bologna et al. (2002) and Bologna and Grigolini (2009). These authors studied quantum systems similar to the “quantum-like” system of Section 4 of Supplementary Material, with two states |1〉 and |2〉 exchanging the population through a non-Markovian equation. They found that although the singular stochastic trajectories analyzed using the DEA method yield the scaling δ=1/(μ−1) , their time-convoluted equation yields δ=(4−μ)/2 .

It is important to note that the time-convoluted equations of Bologna et al. (2002) and Bologna and Grigolini (2009) are a generalization of the standard Fokker–Planck equation, replacing the operator K with a second-order derivative $d^{2}/dx^{2}$ , where x is the diffusion variable. In other words, the time-convoluted equations of Bologna et al. (2002) and Bologna and Grigolini (2009) are equivalent to transforming Equation 24 into a diffusional process. The stochastic variable r(t) adopted to interpret the non-Markov master equation as a stochastic process is a vanishing variable hosting rare values equal to 1. The time distance between two consecutive non-vanishing values is given by a waiting time distribution density ψ(τ) , with the structure as Equation 2. As a consequence, according to the prescriptions of Grigolini et al. (2001), DEA should yield δ=(μ−1) for μ<2 and δ=1/(μ−1) for μ>2 . The conflict between trajectories and densities discussed by Bologna et al. (2002) and Bologna and Grigolini (2009) seem to be a consequence of the fact the theory of quantum mechanics yields the scaling δ=(4−μ)/2 , and the emergence of the trajectory scaling requires a sort of collapse of the wave function.

### 5.4 Meditation

Finally, returning to the influence of meditation on physiological processes (Tuladhar et al., 2018), the use of NVGM does not help settle the paradox that meditation has the effect of reducing the scaling value δ rather than increasing it, considering δ a measure of cognition as assumed by Allegrini et al. (2009). The paradoxical effect of meditation making δ decreasing rather than increasing is also in conflict with the origin of 1/f noise. As proved by Lukovic and Grigolini (2008), 1/f noise is closely connected to the action of crucial events. The power spectrum P(f) is proportional to $1/f^{\beta }$ , and β and μ are connected by the Equation 35:

β=3−μ (35)

In this framework, μ=2 corresponds to ideal 1/f noise. This study supports the choice δ=μ−1 for μ<2 and δ=1/(μ−1) for μ>2 , implying that β=1 can be reached either by increasing μ when μ<2 or by decreasing μ when μ>2 . In both cases, the role of meditation should make δ increase.

A recent study (Pascarella et al., 2025), which emphasizes the role of meditation in modulating the slope of 1/f noise, is in agreement with the findings of Tuladhar et al. (2018). While Tuladhar et al. (2018) investigated the effect of meditation on heartbeat dynamics and Allegrini et al. (2009) focused on EEG signals, another important contribution is provided by Schizas et al. (2025), where the concept of complexity synchronization suggests that meditation should increase the scaling exponent δ in physiological processes, independently of whether they refer to EEG or cardiac signals.

### 5.5 Network physiology

According to Ivanov (2021), “….the new conceptual framework of Network Physiology focuses on the coordination and network interactions among diverse organ systems and sub-systems as a hallmark of physiologic state and function.” This study focuses on the influence of meditation on the dynamics of heart and consequently on the interaction between the brain and heart. Ivanov (2021) also emphasized that interdisciplinarity is a fundamental property of network physiology by the following remarks: “These synergetic effects certainly establish Network Physiology as a new field in the landscape of contemporary biomedical and interdisciplinary research. Understanding the relationship, conceptual difference, the broad horizon and impact of Network Physiology is important to facilitate an active and productive dialog among physicists, biologists, physiologists, neuroscientists and medical clinicians.” Section 3 leads to the important conclusion that meditation makes δ decrease, thereby suggesting further work to do to benefit from interdisciplinarity of network physiology. This interdisciplinary research work is illustrated in the next subsection.

### 5.6 Proposal for future research work

This paper settles the confusion generated by the use of NVGM to define the complexity of physiological processes, making it clear that meditation has the paradoxical effect of forcing physiological processes to depart from the ideal condition of maximal cognition, μ=2 . In conclusion, the relationship between cognition and meditation remains an open and stimulating research topic requiring a deeper understanding of the influence of meditation on the power index μ of the crucial events. The results presented in this study contribute to this discussion with the main suggestion of observing the physiological effects of meditation, not only during meditation but also after meditation. Notably, little attention has been devoted to the immediate post-meditation brain dynamics, which may involve a transition toward scaling values larger than those observed prior to meditation. This phenomenon is particularly relevant to network physiology, where such scaling transitions could reflect coordinated changes across brain–heart, potentially indicating altered inter-regional connectivity and synchronization patterns characteristic of meditative states.

As a further proposal for future research work, the effects of meditation on brain dynamics will be investigated to contribute the debate generated by Linkenkaer-Hansen et al. (2001) with special attention to the connection between criticality and cognition (Müller et al., 2025). Moreover, contributions will be made to the discussion initiated by Linkenkaer-Hansen et al. (2001) and to the therapeutic treatment of mental diseases, keeping in mind that losing crucial events while maintaining a large scaling may be an important form of pathology that the NVGM cannot allow us to detect without using the main results of this paper.

## Data Availability

Publicly available datasets were analyzed in this study. This data can be found here: https://doi.org/10.13026/kah4-0w24 (Healthy), https://doi.org/10.13026/cec2-9w70 (CHF) and https://doi.org/10.1016/S0167-5273(99)00066-2 (Meditation).
