# Supplementary material for: From visibility graphs to cognition
Source: Front Netw Physiol. 2026 May 29;6:1830261. doi: 10.3389/fnetp.2026.1830261 (PMC13259724; doi:10.3389/fnetp.2026.1830261)
Supplement: Supplementary file 1 [file DataSheet1.pdf]

Supplementary Material

1 HEALTHY SUBJECTS (BUT QDB)

Table S1 illustrates the analysis of healthy subjects done with DEA, with and without stripes, and with VGM. In this case the use of stripes does not yield a significant reduction of δ, suggesting that the dominant component hosts crucial events.

Table S1: Scaling exponent δ with stripes, the crucial event index $\mu = 1 + 1/\delta$, the scaling exponent δ without stripes, the Hurst exponent $H = (5-\gamma)/2$ from VGM, and the Hurst exponent $H = (4-\mu)/2$ from crucial events for all 15 healthy subjects from the BUT QDB database Nemcova et al. (2020).

Subject	δ with stripes	μ	δ without stripes	$H = \frac{5-\gamma}{2}$	$H = \frac{4-\mu}{2}$
105001	0.92	2.09	0.95	0.93	0.96
104001	0.85	2.18	0.86	0.87	0.91
103001	0.90	2.11	0.92	0.96	0.95
100001	0.92	2.09	0.96	0.94	0.96
100002	0.93	2.08	0.94	0.94	0.96
111001	0.94	2.06	0.95	0.99	0.97
113001	0.92	2.09	0.94	0.94	0.96
114001	0.91	2.10	0.95	0.92	0.95
115001	0.86	2.16	0.90	0.89	0.92
118001	0.86	2.16	0.88	0.90	0.92
126001	0.89	2.12	0.93	0.98	0.94
125001	0.89	2.12	0.94	0.93	0.94
124001	0.88	2.14	0.94	0.90	0.93
123001	0.90	2.11	0.93	0.91	0.95
121001	0.94	2.06	0.95	0.98	0.97

2 CHF SUBJECTS (MUSIC DATABASE)

Table S2 applies the same analysis as that adopted for healthy subjects to CHF subjects. The result of this analysis confirms the benefit of the joint use of DEA, with and without stripes, and VGM. This is a compelling indication that the pathology-induced disappearance of crucial events does not significantly change the value of the scaling.

Table S2: Scaling exponent δ with stripes, the crucial event index $\mu = 1 + 1/\delta$, the scaling exponent δ without stripes, the Hurst exponent $H = (5-\gamma)/2$ from VGM, and the Hurst exponent $H = (4-\mu)/2$ from crucial events for all 19 CHF subjects from the MUSIC database Martin-Yebra et al. (2024).

Subject	δ with stripes	μ	δ without stripes	$H = \frac{5-\gamma}{2}$	$H = \frac{4-\mu}{2}$
P0001	0.82	2.22	0.91	0.88	0.89
P0002	0.83	2.20	0.93	0.97	0.90
P0003	0.76	2.32	0.89	0.83	0.84
P0004	0.86	2.16	0.95	0.97	0.92
P0005	0.86	2.16	0.94	0.98	0.92
P0006	0.89	2.12	0.90	0.99	0.94
P0007	0.80	2.25	0.93	0.59	0.88
P0010	0.83	2.20	0.94	1.00	0.90
P0015	0.75	2.33	0.96	0.99	0.84
P0017	0.86	2.16	0.94	0.94	0.92
P0150	0.83	2.20	0.93	0.94	0.90
P0065	0.78	2.28	0.93	0.98	0.86
P0086	0.86	2.16	0.94	0.99	0.92
P0110	0.79	2.27	0.93	0.89	0.87
P0147	0.82	2.22	0.92	0.96	0.89
P0253	0.83	2.20	0.94	0.86	0.90
P0099	0.85	2.18	0.95	0.98	0.91
P0138	0.82	2.22	0.93	0.97	0.89
P0755	0.71	2.41	0.87	0.97	0.80

3 VISIBILITY GRAPH RESULTS FOR CHI-MEDITATION SUBJECTS

Table S3 summarizes the power-law exponent $\gamma(H)$ before and during Chi-meditation. The consistent increase in $\gamma(H)$ during meditation corresponds to the decrease of Hurst exponent H through the relation $H = (5 - \gamma)/2$. The δ scaling values before and during meditation presented in the table are derived from the earlier work of Tuladhar et al. (2018).

Table S3: Power-law exponent $\gamma(H)$ and scaling index δ obtained from VGM before and during Chi-meditation Peng et al. (1999)

Subject	$\gamma(H)$ Before	δ_{Before}	$\gamma(H)$ During	δ_{During}
Chi-1	3.24	0.68	3.86	0.63
Chi-2	2.97	0.78	3.15	0.65
Chi-3	3.10	0.75	3.55	0.63
Chi-4	2.87	0.77	3.44	0.65
Chi-5	3.23	0.68	3.47	0.63
Chi-6	3.02	0.75	3.60	0.64
Chi-7	2.96	0.80	3.00	0.80
Chi-8	3.11	0.72	3.39	0.63

REFERENCES

Nemcova A, Smisek R, Opravilová K, Vitek M, Smital L, Maršánová L. Brno University of Technology ECG Quality Database (BUT QDB). PhysioNet (2020). doi:10.13026/kah4-0w24. Version 1.0.0.

Martin-Yebra A, Martínez JP, Laguna P. MUSIC (Sudden Cardiac Death in Chronic Heart Failure). PhysioNet (2024). doi:10.13026/cec2-9w70. Version 1.0.0.

Tuladhar R, Bohara G, Grigolini P, West BJ. Meditation-induced coherence and crucial events. Front. Physiol. 9 (2018) 626. doi:10.3389/fphys.2018.00626.

Peng CK, Mietus JE, Liu Y, Khalsa G, Douglas PS, Benson H, et al. Exaggerated heart rate oscillations during two meditation techniques. International journal of cardiology 70 (1999) 101–107.
